# 2-[(3,5-Di-*tert*-butyl-4-hy­droxy­benz­yl)sulfan­yl]benzoic acid

**DOI:** 10.1107/S1600536810023731

**Published:** 2010-06-26

**Authors:** Abeer A. Alhadi, Hamid Khaledi, Hapipah Mohd Ali, Marilyn M. Olmstead

**Affiliations:** a Department of Chemistry, University of Malaya, 50603 Kuala Lumpur, Malaysia; b Department of Chemistry, University of California, One Shields Avenue, Davis, CA 95616, USA

## Abstract

In the title compound, C_22_H_28_O_3_S, the dihedral angle between the two aromatic rings is 80.56 (6)°. The hy­droxy group is shielded by the two sterically hindered *tert*-butyl groups and therefore is not involved in any hydrogen bonding. The C—O—H fragment is coplanar with the aromatic ring, the dihedral angle between them being 7(5)°. In the crystal structure, pairs of mol­ecules are hydrogen bonded across crystallographic centers of symmetry.

## Related literature

For a similar structure based on nicotinic acid, see: Mansor *et al.* (2008 ▶).
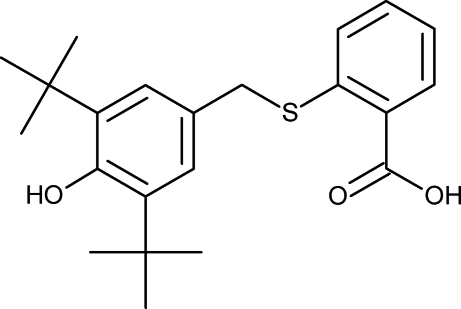


## Experimental

### 

#### Crystal data

C_22_H_28_O_3_S
*M* *_r_* = 372.50
Monoclinic, 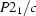

*a* = 18.1496 (4) Å
*b* = 5.6863 (1) Å
*c* = 20.2159 (4) Å
β = 101.172 (1)°
*V* = 2046.83 (7) Å^3^
*Z* = 4
Mo *K*α radiation
μ = 0.18 mm^−1^
*T* = 296 K
0.35 × 0.28 × 0.18 mm

#### Data collection

Bruker APEXII CCD diffractometer
Absorption correction: multi-scan (*SADABS*; Sheldrick, 1996 ▶) *T*_min_ = 0.941, *T*_max_ = 0.969
15313 measured reflections
3610 independent reflections
2477 reflections with *I* > 2σ(*I*)
*R*_int_ = 0.041

#### Refinement

*R*[*F* ^2^ > 2σ(*F* ^2^)] = 0.045
*wR*(*F* ^2^) = 0.123
*S* = 1.02
3610 reflections
247 parameters
1 restraint
H atoms treated by a mixture of independent and constrained refinement
Δρ_max_ = 0.32 e Å^−3^
Δρ_min_ = −0.17 e Å^−3^

### 

Data collection: *APEX2* (Bruker, 2007 ▶); cell refinement: *SAINT* (Bruker, 2007 ▶); data reduction: *SAINT*; program(s) used to solve structure: *SHELXS97* (Sheldrick, 2008 ▶); program(s) used to refine structure: *SHELXL97* (Sheldrick, 2008 ▶); molecular graphics: *X-SEED* (Barbour, 2001 ▶); software used to prepare material for publication: *SHELXL97* and *publCIF* (Westrip, 2010 ▶).

## Supplementary Material

Crystal structure: contains datablocks I, global. DOI: 10.1107/S1600536810023731/pk2249sup1.cif

Structure factors: contains datablocks I. DOI: 10.1107/S1600536810023731/pk2249Isup2.hkl

Additional supplementary materials:  crystallographic information; 3D view; checkCIF report

## Figures and Tables

**Table 1:** Hydrogen-bond geometry (Å, °)

*D*—H⋯*A*	*D*—H	H⋯*A*	*D*⋯*A*	*D*—H⋯*A*
O1—H1⋯O2^i^	0.84 (2)	1.83 (2)	2.661 (2)	178 (4)

Symmetry code: (i) 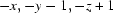.

**Fig. 1.**
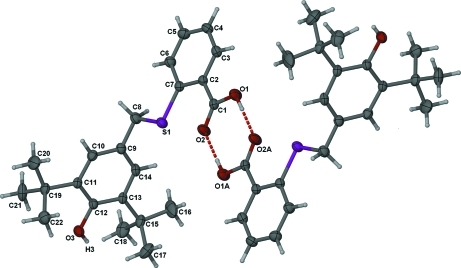
A perspective drawing of two molecules of the title compound showing dimerization through intermolecular H-bonds. Displacement ellipsoids are drawn at the 30% probability level.

## supplementary crystallographic information

### Comment

Interest in the title compound stems from its use as an antioxidant. The hydroxyl group resides in a sterically encumbered pocket provided by the 3,5-di *tert*-butyl groups. Consequently, it is capable of forming a stable phenoxy radical. The position of the hydroxyl hydrogen atom is interesting since it has little space. The final difference map shows the hydrogen atom as the largest peak, and it is nearly in the plane of the phenyl ring. The hydrogen (H3) was refined in this position with U(H3) = 1.5*U*_eq_(O3); however, there are unavoidable short contacts, the shortest being 1.83 Å to one of the methyl H atoms (H18A). In the structure of the similar compound (Mansor *et al.*, 2008), the hydrogen atom was placed in a "chemically sensible" position, perpendicular to the phenyl group, that yielded a more distant contact (the shortest is 2.05 Å to one of the methyl H atoms). However, we have examined the difference map for this structure and found that the peak of maximum electron density is not perpendicular to the phenyl ring and clearly lies in the plane of the phenyl ring, as it does in the title compound. Upon free refinement, the shortest contact from the hydroxyl hydrogen to the nearest methyl hydrogen is 1.80 Å. Therefore, we would like to suggest that these structures do, indeed, display short H···H contacts that bear further investigation, preferably at very low (*i.e.* He cryostat) temperatures.

### Experimental

Thiosalicylic acid (0.154 g, 1 mmol), 2,6-di-*t*-butylphenol (2.00 g, 1 mmol) and paraformaldehyde (0.291 g,1 mmol) were ground into a homogenous powder and to this was added di-*n*-butylamine (0.09 ml). The slurry was heated to 353 K for 2.5 h, then cooled to 323 K, ethanol (20 ml) was added and stirred for 1 h. The mixture was then acidified to pH= 5–5.5 by using 1.5% hydrochloric acid solution. The slurry was filtered and the filtrate was kept in the dark for 5 days whereupon the colorless crystals of the title compound were obtained.

### Refinement

The C-bound hydrogen atoms were placed at calculated positions (C—H 0.93–0.97 Å) and were treated as riding on their parent atoms with U(H) set to 1.2–1.5 *U*_eq_(C). The carboxylic acid H-atom was located in a difference Fourier map and was refined with a distance restraint of O—H 0.82 (2) Å. The hydroxyl H-atom was located in a difference Fourier map and was refined with U(H) set to 1.5*U*_eq_(O3).

### Crystal data

**Table:**

C_22_H_28_O_3_S	*F*(000) = 800
*M**_r_* = 372.50	*D*_x_ = 1.209 Mg m^−^^3^
Monoclinic, *P*2_1_/*c*	Mo *K*α radiation, λ = 0.71073 Å
Hall symbol: -P 2ybc	Cell parameters from 2170 reflections
*a* = 18.1496 (4) Å	θ = 2.2–21.4°
*b* = 5.6863 (1) Å	µ = 0.18 mm^−^^1^
*c* = 20.2159 (4) Å	*T* = 296 K
β = 101.172 (1)°	Block, colourless
*V* = 2046.83 (7) Å^3^	0.35 × 0.28 × 0.18 mm
*Z* = 4	

### Data collection

**Table:**

Bruker APEXII CCD diffractometer	3610 independent reflections
Radiation source: fine-focus sealed tube	2477 reflections with *I* > 2σ(*I*)
graphite	*R*_int_ = 0.041
φ and ω scans	θ_max_ = 25.0°, θ_min_ = 2.1°
Absorption correction: multi-scan (*SADABS*; Sheldrick, 1996)	*h* = −21→21
*T*_min_ = 0.941, *T*_max_ = 0.969	*k* = −6→6
15313 measured reflections	*l* = −24→24

### Refinement

**Table:**

Refinement on *F*^2^	Primary atom site location: structure-invariant direct methods
Least-squares matrix: full	Secondary atom site location: difference Fourier map
*R*[*F*^2^ > 2σ(*F*^2^)] = 0.045	Hydrogen site location: inferred from neighbouring sites
*wR*(*F*^2^) = 0.123	H atoms treated by a mixture of independent and constrained refinement
*S* = 1.01	*w* = 1/[σ^2^(*F*_o_^2^) + (0.0518*P*)^2^ + 0.6688*P*] where *P* = (*F*_o_^2^ + 2*F*_c_^2^)/3
3610 reflections	(Δ/σ)_max_ < 0.001
247 parameters	Δρ_max_ = 0.32 e Å^−^^3^
1 restraint	Δρ_min_ = −0.17 e Å^−^^3^

### Special details

**Table:**

Geometry. All e.s.d.'s (except the e.s.d. in the dihedral angle between two l.s. planes) are estimated using the full covariance matrix. The cell e.s.d.'s are taken into account individually in the estimation of e.s.d.'s in distances, angles and torsion angles; correlations between e.s.d.'s in cell parameters are only used when they are defined by crystal symmetry. An approximate (isotropic) treatment of cell e.s.d.'s is used for estimating e.s.d.'s involving l.s. planes.
Refinement. Refinement of *F*^2^ against ALL reflections. The weighted *R*-factor *wR* and goodness of fit *S* are based on *F*^2^, conventional *R*-factors *R* are based on *F*, with *F* set to zero for negative *F*^2^. The threshold expression of *F*^2^ > σ(*F*^2^) is used only for calculating *R*-factors(gt) *etc*. and is not relevant to the choice of reflections for refinement. *R*-factors based on *F*^2^ are statistically about twice as large as those based on *F*, and *R*- factors based on ALL data will be even larger.

### Fractional atomic coordinates and isotropic or equivalent isotropic displacement parameters (Å^2^)

**Table:**

	*x*	*y*	*z*	*U*_iso_*/*U*_eq_	
S1	0.08318 (3)	0.07380 (12)	0.38838 (3)	0.0556 (2)	
O1	−0.08616 (10)	−0.4044 (4)	0.44667 (10)	0.0713 (6)	
H1	−0.0691 (18)	−0.500 (5)	0.4773 (14)	0.107*	
O2	0.03143 (10)	−0.2849 (3)	0.45796 (9)	0.0722 (6)	
H3	0.437 (2)	0.532 (6)	0.4727 (18)	0.108*	
O3	0.42604 (9)	0.4524 (3)	0.44311 (8)	0.0570 (5)	
C1	−0.03435 (13)	−0.2647 (5)	0.43156 (12)	0.0511 (6)	
C2	−0.06278 (12)	−0.0867 (4)	0.37992 (11)	0.0455 (5)	
C3	−0.13987 (13)	−0.0776 (5)	0.35422 (12)	0.0560 (6)	
H3A	−0.1715	−0.1827	0.3704	0.067*	
C4	−0.17013 (14)	0.0820 (5)	0.30591 (13)	0.0602 (7)	
H4	−0.2217	0.0869	0.2898	0.072*	
C5	−0.12312 (14)	0.2351 (5)	0.28141 (13)	0.0617 (7)	
H5	−0.1431	0.3419	0.2478	0.074*	
C6	−0.04738 (14)	0.2318 (5)	0.30594 (12)	0.0555 (6)	
H6	−0.0168	0.3374	0.2887	0.067*	
C7	−0.01457 (12)	0.0736 (4)	0.35630 (11)	0.0453 (5)	
C8	0.11580 (13)	0.3226 (4)	0.34536 (13)	0.0551 (6)	
H8A	0.1071	0.2936	0.2972	0.066*	
H8B	0.0887	0.4637	0.3532	0.066*	
C9	0.19819 (13)	0.3541 (4)	0.37237 (12)	0.0476 (6)	
C10	0.25124 (13)	0.2638 (4)	0.33862 (11)	0.0482 (6)	
H10	0.2348	0.1782	0.2993	0.058*	
C11	0.32779 (12)	0.2952 (4)	0.36066 (11)	0.0438 (5)	
C12	0.34993 (12)	0.4227 (4)	0.42063 (11)	0.0424 (5)	
C13	0.29827 (13)	0.5169 (4)	0.45709 (11)	0.0448 (5)	
C14	0.22284 (13)	0.4794 (4)	0.43102 (12)	0.0506 (6)	
H14	0.1874	0.5410	0.4539	0.061*	
C15	0.32235 (14)	0.6534 (4)	0.52338 (12)	0.0545 (6)	
C16	0.25557 (18)	0.7412 (8)	0.55221 (18)	0.1132 (14)	
H16A	0.2253	0.8436	0.5202	0.170*	
H16B	0.2735	0.8257	0.5933	0.170*	
H16C	0.2259	0.6096	0.5613	0.170*	
C17	0.3697 (2)	0.4965 (6)	0.57768 (14)	0.0901 (10)	
H17A	0.3393	0.3695	0.5886	0.135*	
H17B	0.3876	0.5883	0.6174	0.135*	
H17C	0.4117	0.4342	0.5609	0.135*	
C18	0.36869 (19)	0.8726 (5)	0.51428 (16)	0.0828 (9)	
H18A	0.4140	0.8260	0.5000	0.124*	
H18B	0.3812	0.9555	0.5563	0.124*	
H18C	0.3399	0.9734	0.4808	0.124*	
C19	0.38448 (14)	0.1959 (4)	0.31979 (11)	0.0518 (6)	
C20	0.34466 (17)	0.0547 (6)	0.25871 (14)	0.0856 (10)	
H20A	0.3111	0.1559	0.2290	0.128*	
H20B	0.3166	−0.0712	0.2736	0.128*	
H20C	0.3813	−0.0092	0.2352	0.128*	
C21	0.42732 (18)	0.3964 (5)	0.29384 (16)	0.0828 (10)	
H21A	0.4538	0.4857	0.3313	0.124*	
H21B	0.3925	0.4970	0.2651	0.124*	
H21C	0.4625	0.3326	0.2688	0.124*	
C22	0.43946 (16)	0.0255 (5)	0.36205 (14)	0.0700 (8)	
H22A	0.4747	−0.0302	0.3358	0.105*	
H22B	0.4123	−0.1054	0.3754	0.105*	
H22C	0.4660	0.1050	0.4015	0.105*	

### Atomic displacement parameters (Å^2^)

**Table:**

	*U*^11^	*U*^22^	*U*^33^	*U*^12^	*U*^13^	*U*^23^
S1	0.0401 (4)	0.0669 (4)	0.0595 (4)	−0.0046 (3)	0.0088 (3)	0.0124 (3)
O1	0.0490 (11)	0.0898 (15)	0.0741 (13)	−0.0145 (10)	0.0098 (9)	0.0283 (11)
O2	0.0433 (11)	0.0890 (14)	0.0831 (13)	−0.0060 (9)	0.0095 (9)	0.0368 (11)
O3	0.0446 (10)	0.0721 (12)	0.0559 (11)	−0.0047 (8)	0.0137 (8)	−0.0186 (9)
C1	0.0431 (15)	0.0646 (16)	0.0484 (14)	−0.0046 (12)	0.0156 (11)	0.0040 (12)
C2	0.0401 (13)	0.0562 (14)	0.0413 (12)	0.0000 (11)	0.0108 (10)	−0.0043 (11)
C3	0.0454 (15)	0.0684 (17)	0.0564 (15)	−0.0010 (12)	0.0152 (12)	−0.0016 (14)
C4	0.0394 (14)	0.0803 (19)	0.0595 (16)	0.0066 (13)	0.0060 (12)	−0.0020 (15)
C5	0.0558 (17)	0.0677 (18)	0.0595 (16)	0.0139 (14)	0.0058 (13)	0.0074 (14)
C6	0.0488 (15)	0.0601 (16)	0.0574 (15)	0.0003 (12)	0.0102 (12)	0.0096 (13)
C7	0.0415 (13)	0.0538 (14)	0.0420 (12)	0.0015 (11)	0.0112 (10)	−0.0032 (11)
C8	0.0468 (15)	0.0589 (16)	0.0610 (16)	−0.0042 (12)	0.0136 (12)	0.0061 (12)
C9	0.0458 (14)	0.0485 (14)	0.0505 (14)	−0.0057 (11)	0.0142 (11)	0.0037 (11)
C10	0.0532 (15)	0.0498 (14)	0.0424 (13)	−0.0057 (11)	0.0112 (11)	−0.0051 (11)
C11	0.0470 (14)	0.0464 (13)	0.0405 (12)	−0.0016 (10)	0.0151 (10)	−0.0006 (10)
C12	0.0410 (13)	0.0451 (13)	0.0435 (12)	−0.0036 (10)	0.0139 (10)	−0.0015 (10)
C13	0.0505 (14)	0.0458 (13)	0.0421 (12)	−0.0036 (10)	0.0190 (11)	−0.0026 (10)
C14	0.0503 (15)	0.0532 (15)	0.0543 (15)	−0.0017 (11)	0.0254 (12)	−0.0018 (12)
C15	0.0667 (17)	0.0565 (15)	0.0455 (14)	−0.0068 (12)	0.0235 (12)	−0.0099 (12)
C16	0.088 (2)	0.158 (4)	0.105 (3)	−0.013 (2)	0.047 (2)	−0.074 (3)
C17	0.128 (3)	0.094 (2)	0.0485 (17)	−0.005 (2)	0.0170 (18)	−0.0055 (16)
C18	0.106 (3)	0.071 (2)	0.076 (2)	−0.0196 (17)	0.0289 (18)	−0.0233 (16)
C19	0.0579 (15)	0.0574 (15)	0.0440 (14)	0.0023 (12)	0.0197 (11)	−0.0077 (12)
C20	0.084 (2)	0.114 (3)	0.0609 (18)	0.0135 (18)	0.0185 (16)	−0.0377 (18)
C21	0.101 (2)	0.080 (2)	0.086 (2)	0.0036 (17)	0.0625 (19)	0.0039 (17)
C22	0.0738 (19)	0.0719 (19)	0.0671 (18)	0.0182 (15)	0.0205 (15)	−0.0081 (14)

### Geometric parameters (Å, °)

**Table:**

S1—C7	1.767 (2)	C13—C14	1.385 (3)
S1—C8	1.819 (2)	C13—C15	1.537 (3)
O1—C1	1.311 (3)	C14—H14	0.9300
O1—H1	0.837 (18)	C15—C16	1.527 (4)
O2—C1	1.215 (3)	C15—C18	1.535 (4)
O3—C12	1.378 (3)	C15—C17	1.541 (4)
O3—H3	0.75 (3)	C16—H16A	0.9600
C1—C2	1.474 (3)	C16—H16B	0.9600
C2—C3	1.396 (3)	C16—H16C	0.9600
C2—C7	1.409 (3)	C17—H17A	0.9600
C3—C4	1.368 (3)	C17—H17B	0.9600
C3—H3A	0.9300	C17—H17C	0.9600
C4—C5	1.377 (4)	C18—H18A	0.9600
C4—H4	0.9300	C18—H18B	0.9600
C5—C6	1.368 (3)	C18—H18C	0.9600
C5—H5	0.9300	C19—C22	1.528 (3)
C6—C7	1.402 (3)	C19—C21	1.529 (4)
C6—H6	0.9300	C19—C20	1.532 (4)
C8—C9	1.500 (3)	C20—H20A	0.9600
C8—H8A	0.9700	C20—H20B	0.9600
C8—H8B	0.9700	C20—H20C	0.9600
C9—C14	1.381 (3)	C21—H21A	0.9600
C9—C10	1.382 (3)	C21—H21B	0.9600
C10—C11	1.386 (3)	C21—H21C	0.9600
C10—H10	0.9300	C22—H22A	0.9600
C11—C12	1.403 (3)	C22—H22B	0.9600
C11—C19	1.546 (3)	C22—H22C	0.9600
C12—C13	1.406 (3)		
			
C7—S1—C8	102.81 (11)	C16—C15—C18	105.9 (3)
C1—O1—H1	113 (2)	C16—C15—C13	112.7 (2)
C12—O3—H3	115 (3)	C18—C15—C13	112.0 (2)
O2—C1—O1	121.9 (2)	C16—C15—C17	107.0 (3)
O2—C1—C2	123.7 (2)	C18—C15—C17	108.2 (2)
O1—C1—C2	114.4 (2)	C13—C15—C17	110.7 (2)
C3—C2—C7	119.6 (2)	C15—C16—H16A	109.5
C3—C2—C1	118.5 (2)	C15—C16—H16B	109.5
C7—C2—C1	121.9 (2)	H16A—C16—H16B	109.5
C4—C3—C2	121.7 (2)	C15—C16—H16C	109.5
C4—C3—H3A	119.2	H16A—C16—H16C	109.5
C2—C3—H3A	119.2	H16B—C16—H16C	109.5
C3—C4—C5	119.0 (2)	C15—C17—H17A	109.5
C3—C4—H4	120.5	C15—C17—H17B	109.5
C5—C4—H4	120.5	H17A—C17—H17B	109.5
C6—C5—C4	120.7 (2)	C15—C17—H17C	109.5
C6—C5—H5	119.7	H17A—C17—H17C	109.5
C4—C5—H5	119.7	H17B—C17—H17C	109.5
C5—C6—C7	121.9 (2)	C15—C18—H18A	109.5
C5—C6—H6	119.1	C15—C18—H18B	109.5
C7—C6—H6	119.1	H18A—C18—H18B	109.5
C6—C7—C2	117.2 (2)	C15—C18—H18C	109.5
C6—C7—S1	121.41 (18)	H18A—C18—H18C	109.5
C2—C7—S1	121.41 (18)	H18B—C18—H18C	109.5
C9—C8—S1	108.23 (17)	C22—C19—C21	110.1 (2)
C9—C8—H8A	110.1	C22—C19—C20	105.9 (2)
S1—C8—H8A	110.1	C21—C19—C20	107.8 (2)
C9—C8—H8B	110.1	C22—C19—C11	111.29 (19)
S1—C8—H8B	110.1	C21—C19—C11	110.3 (2)
H8A—C8—H8B	108.4	C20—C19—C11	111.3 (2)
C14—C9—C10	118.3 (2)	C19—C20—H20A	109.5
C14—C9—C8	120.6 (2)	C19—C20—H20B	109.5
C10—C9—C8	121.1 (2)	H20A—C20—H20B	109.5
C9—C10—C11	123.0 (2)	C19—C20—H20C	109.5
C9—C10—H10	118.5	H20A—C20—H20C	109.5
C11—C10—H10	118.5	H20B—C20—H20C	109.5
C10—C11—C12	116.5 (2)	C19—C21—H21A	109.5
C10—C11—C19	120.7 (2)	C19—C21—H21B	109.5
C12—C11—C19	122.8 (2)	H21A—C21—H21B	109.5
O3—C12—C11	116.68 (19)	C19—C21—H21C	109.5
O3—C12—C13	120.53 (19)	H21A—C21—H21C	109.5
C11—C12—C13	122.8 (2)	H21B—C21—H21C	109.5
C14—C13—C12	116.9 (2)	C19—C22—H22A	109.5
C14—C13—C15	120.2 (2)	C19—C22—H22B	109.5
C12—C13—C15	122.9 (2)	H22A—C22—H22B	109.5
C9—C14—C13	122.5 (2)	C19—C22—H22C	109.5
C9—C14—H14	118.7	H22A—C22—H22C	109.5
C13—C14—H14	118.7	H22B—C22—H22C	109.5

### Hydrogen-bond geometry (Å, °)

**Table:**

*D*—H···*A*	*D*—H	H···*A*	*D*···*A*	*D*—H···*A*
O1—H1···O2^i^	0.84 (2)	1.83 (2)	2.661 (2)	178 (4)

Symmetry codes: (i) −*x*, −*y*−1, −*z*+1.
